# Additional Neural Matrix Factorization model for computational drug repositioning

**DOI:** 10.1186/s12859-019-2983-2

**Published:** 2019-08-14

**Authors:** Xinxing Yang, lbrahim Zamit, Yu Liu, Jieyue He

**Affiliations:** School of Computer Science and Engineering, Key Lab of Computer Network & Information Integration, MOE, Southeast University, Nanjing, 210018 China

**Keywords:** Drug repositioning, Data mining, Matrix factorization, Neural network

## Abstract

**Background:**

Computational drug repositioning, which aims to find new applications for existing drugs, is gaining more attention from the pharmaceutical companies due to its low attrition rate, reduced cost, and shorter timelines for novel drug discovery. Nowadays, a growing number of researchers are utilizing the concept of recommendation systems to answer the question of drug repositioning. Nevertheless, there still lie some challenges to be addressed: 1) Learning ability deficiencies; the adopted model cannot learn a higher level of drug-disease associations from the data. 2) Data sparseness limits the generalization ability of the model. 3)Model is easy to overfit if the effect of negative samples is not taken into consideration.

**Results:**

In this study, we propose a novel method for computational drug repositioning, Additional Neural Matrix Factorization (ANMF). The ANMF model makes use of drug-drug similarities and disease-disease similarities to enhance the representation information of drugs and diseases in order to overcome the matter of data sparsity. By means of a variant version of the autoencoder, we were able to uncover the hidden features of both drugs and diseases. The extracted hidden features will then participate in a collaborative filtering process by incorporating the Generalized Matrix Factorization (GMF) method, which will ultimately give birth to a model with a stronger learning ability. Finally, negative sampling techniques are employed to strengthen the training set in order to minimize the likelihood of model overfitting. The experimental results on the Gottlieb and Cdataset datasets show that the performance of the ANMF model outperforms state-of-the-art methods.

**Conclusions:**

Through performance on two real-world datasets, we believe that the proposed model will certainly play a role in answering to the major challenge in drug repositioning, which lies in predicting and choosing new therapeutic indications to prospectively test for a drug of interest.

## Background

Traditional new drug design and discovery are an expensive, time-consuming and high-risk process. For instance, it takes at least 10–15 years, and an estimated budget of 8–10 billion dollars to develop and bring a new drug to the market [1, 2]. Since the 1990s, the annual quota of new drugs approved by the US Food and Drug Administration (FDA) has been declining. Meanwhile, biopharmaceutical companies continue to increase their investments in new drug design and discovery [3], which implies that new drugs are becoming more and more expensive. And drugs designed for specific targets often have unperceivable side effects, about 90% of experimental drugs fail to pass the first phase of clinical trials [4]. The process of developing innovative drugs remains expensive, time-consuming and full of uncertainty. In light of these challenges, Computational drug repositioning, which aims to find new uses and applications for existing drugs, has become an alternative for the traditional new drug discovery. The drugs approved for sale, which has undergone several rigorous clinical trials are ensured to be safe as they already passed laborious assessments for any unpleasant side effects [5]. Hence, drugs designed according to the new applications are more likely to pass the screening of regulatory authorities [6].

The core of computational drug repositioning is to mine new uses of existing drugs, and treat diseases that are not within its original design. Drug repositioning begins with an accidental discovery of new applications of the original drug. Taking thalidomide as an example [5], the drug was first used as a sedative in Germany, marketed in the United Kingdom as a treatment to nausea and insomnia, and it is also used to relieve pregnancy reactions among pregnant women. First listed in 1956 and banned in 1962, the reintegration of thalidomide again as a drug is attributed to the accidental discovery that it can be used to treat leprosy nodular erythema. Cases of drugs like thalidomide reflect the fact that a single medication can treat multiple diseases. As an essential technology to discover new applications of old drugs, and an efficient way to improve R&D productivity, computational drug repositioning has been receiving a great deal of attention from the biotech and pharmaceutical industries.

In recent years, researchers have explored a variety of computational drug repositioning approaches, such as graph-based methods, matrix factorization based methods, Collaborative filtering etc. In relevance to our inspiration for the presented work in this paper, we will give a broad research overview for related work in the area of computational drug repositioning. The aim is to further clarify the research standing of the proposed model, and showcase our initial setup motivations.

Graph-based models are considered to be the cornerstone of the search recommendation area, used in many fields, such as social networks and search engines to name a few. Based on the provided information, the graph model first constructs a connection diagram between research objects according to certain rules. This diagram can be a directed or undirected graph. In drug repositioning problem, there are at least two types of nodes, drug nodes and disease nodes. The graph model constructs a drug-disease network according to the therapeutic relationships between drugs and diseases. Selecting the appropriate strategy used to estimate the associations is key to the success of the graph model, such as recent distance, public neighbors and other approaches. Li et al. [7] proposed a method based on the “guilt-by-association” notion, which uses all known proteins and drugs to construct nodes- and edges-weighted biological relevant interactome network. The novel network topology features are proposed to characterize interaction pairs, and random forest algorithm is employed to identify potential drug-protein interaction. Chen et al. [8] proposed a method, under the hypothesis that similar drugs often target similar target proteins and the framework of random walk, to predict potential drug–target interactions on a large scale. Wang et al. [9] proposed a method named Heterogeneous Graph Based Inference (HGBI). A heterogeneous drug-target graph, which incorporates known drug-target interactions as well as drug-drug and target-target similarities, is first constructed. Based on this graph, a novel drug and target association prediction technique is inferred. Martinez et al. [10] proposed a new methodology for drug-disease and disease-drug prioritization named DrugNet. Based on a previously developed network-based prioritization method called ProphNet, they were able to build a three-layer heterogeneous network that contained diverse types of elements and interactions. Their findings suggest that DrugNet could be very useful for discovering new drug use cases, and the integration of heterogeneous data would be beneficial to improve the performance of classifiers for the drug repositioning task. Luo et al. [11] proposed a computational method to find novel indications for existing drugs. By applying comprehensive similarity measures, they were able to build a heterogeneous network with known drug-disease interactions. Bi-Random Walk algorithm was then implemented to predict innovative drug-disease associations.

Matrix factorization based models assume that several factors can represent each drug and disease. When drugs and diseases characteristics are consistent in the matrix, it is believed that there is a high correlation between the drug and the disease; that is, the drug may be used to treat the disease. This model decomposes the known drug-disease treatment association matrix into two low-rank drugs and disease potential factor matrices. Usually, the rank of the latent factor matrix is much smaller than the number of drugs or diseases. Matrix factorization technique is widely used in data dimensionality reduction, and recommendation application scenarios. Researchers continue to improve the matrix decomposition model for the drug repositioning task to adapt to the application scenario, as the use of a single feature does not entirely imitate the characteristics of drugs and diseases. Zhang et al. [12] proposed a unified computational platform which presents the task of hypothesis generation for drug repositioning as a constrained nonlinear optimization problem. They utilized a three-layer network approach to explore potential new associations among drugs and diseases with no prior links. Dai et al. [13] based on the idea that association between drug and disease has its evidence in the interactome network of genes. The authors proposed a matrix factorization model, which incorporates the biological information of genomic space interactions for the prediction of novel drug-disease associations. According to the drug-disease relationships, Luo et al. [14] proposed the Drug Repositioning Recommendation System (DRRS) to predict novel interactions for known drugs. This method used the drug similarity and disease similarity to construct a heterogeneous network, which was represented by a drug-disease adjacency matrix. Finally, the drug relocation could be realized by completing the matrix with the use of fast Singular Value Thresholding (SVT) algorithm presented in [15].

Collaborative filtering is commonly used to learn and predict the relationship between users and items in a recommendation system scenario. Lately, some researchers turned to collaborative filtering to tackle the challenge of drug repositioning. Following the same belief as Dai et al. [13], Regenbogen et al. [16] via using a collaborative filtering approach, constructed a relationship matrix comprising drugs, diseases, and genetic information. Non-Negative Matrix Factorization (NMF) technique was then introduced to predict the correlation between drugs and diseases. Zhang et al. [17] proposed the model which uses a neighbor-based collaborative filtering technique to incorporate complex data information for drug-disease relationship prediction.

Nevertheless, the above methods based on recommendation systems are limited in three aspects: insufficient learning ability, data sparsity, and disregarding the effect of negative samples. Matrix factorization models the drug-disease relationship as an inner product of drug latent factors and disease potential factors, which is a linear combination. The combination itself does not take into account the weight relationship between factors, and cannot learn the complex associations between drugs and diseases. In addition, the number of diseases which can be treated by a single medication is small. Similarly, the number of drugs that can be applied to cure the same illness is low as well. As a result, merely relying on drug-disease treatment relationship data cannot adequately reflect the relationship between drugs and diseases.

Moreover, the previously described models ignore the negative sampling technique, and only uses known drug-disease associations. This exclusion may lead to overfitting, and degrades the performance of the model on the test set. Therefore, to overcome the shortcomings mentioned above, we propose an Additional Neural Matrix Factorization (ANMF) model. The ANMF model combines additional auxiliary information, neural network, and matrix factorization to infer novel treatments for diseases.

So as to overcome data sparsity, the ANMF model makes use of drug-drug and disease-disease similarities to enhance the representation information of drugs and diseases. Uncovering the hidden features of both drugs and diseases is made possible by the use of a deep learning technique, Additional Stacked Denoising Autoencoder (ADAE) [18]. The extracted hidden features will then participate in a collaborative filtering process by utilizing the idea of the product operation of the Generalized Matrix Factorization (GMF) method [19]. The GMF product operation introduces neuronal nodes and a nonlinear activation function. Therefore, the model can uncover further nonlinear relationships between drugs and diseases. This procedure will eventually allow us to obtain a model with a greater learning ability. Lastly, with the aim of minimizing the likelihood of model overfitting, negative sampling techniques are employed to strengthen the training set. Compared with the state-of-the-art models, the ANMF model is shown to be more valid. We can summarize the main contributions of this paper as follows:

(1) A novel Additional Neural Matrix Factorization (ANMF) model is proposed for drug repositioning. The model combines deep learning representation with the nonlinear matrix factorization technique, and allows for integration of auxiliary information regarding drugs and diseases during the hidden features extraction process. As follows, a better-hidden relationship between drugs and diseasees can be captured.

(2) The negative sampling technique mentioned in [20] from the field of natural language processing is used to enhance the training set, which reduces the possibility of overfitting. The generalization feature of the model is improved as well.

(3) The ANMF model tested both on the Gottlieb dataset [21] and the Cdataset [14], is assumed to retain its validity as its AUC (Area Under Curve), AUPR (Area Under Precision-Recall Curve) and HR (Hit Ratio) values are superior to that of the state-of-the-art related model’s benchmarks.

The rest of this paper is as constructed as follows: we will introduce the implementation details and principles of the ANMF model in “Methods” section. In “Results” section, the experiments and results of the ANMF model on the Gottlieb dataset and the Cdataset will be discussed. The corresponding discussions are presented in “Discussion” section. The final “Conclusion” section will serve as a summary of our work and a guideline for future ventures.

## Methods

**Fig. 1:**
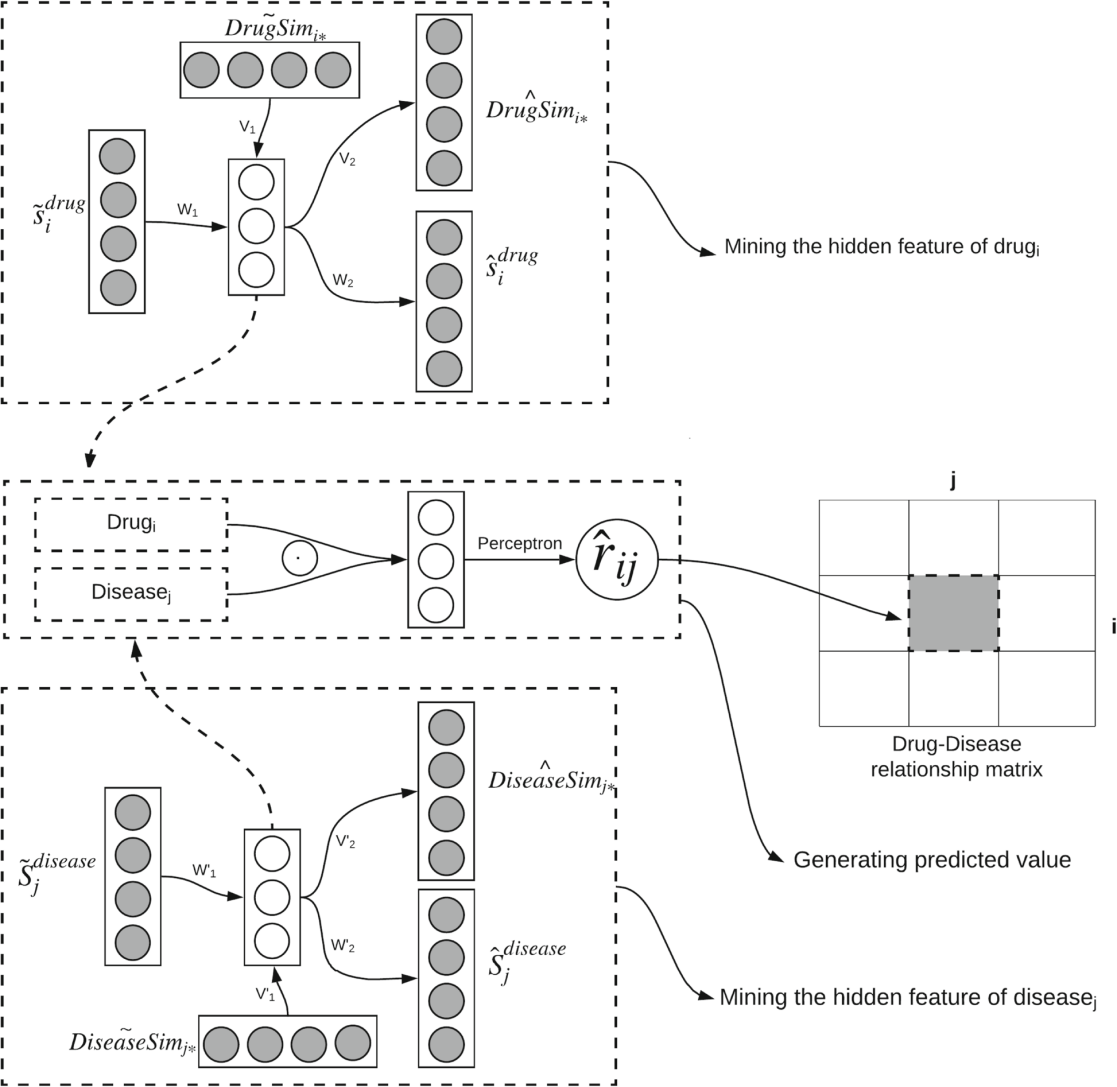
The architecture of the ANMF model

The ANMF model proposed for drug repositioning combines neural network with matrix factorization model, and fuses additional auxiliary information to infer novel treatments for diseases. Figure 1 shows the architecture of the ANMF model.

The upper part of Fig. 1 is the process of mining the hidden feature of drug *i*, where *drug*_*i*_ indicates the hidden feature of drug *i*. The bottom portion is the process of mining the hidden feature of disease *j*, where *disease*_*j*_ indicates the hidden feature of disease *j*. The procedure of mining the hidden features of diseases and drugs is in reality the reconstruction of drug and disease attribute features. This process will be described in detail in “Hidden feature mining” section. The middle part of Fig. 1 shows the elementwise product operation of the extracted *drug*_*i*_ and *disease*_*j*_. Finally, the product result will be inputted into a single layer perceptron to predict the drug-disease relationship. The prediction process will be described thoroughly in “Generate predicted value” section. In “ANMF Learning process” section, we will define the general loss function of the ANMF model, and show how the model can learn the corresponding parameters. Incorporating the negative sampling techniques onto the training set with will be described in the “Defining the number of negative sampling” section.

At present, the field of deep learning is still considered as a “blackbox process”, lacking a set of axiomatic mathematical proof. However, we can proceed from the practical significance of matrix factorization model. The hidden features of drugs store the specific preferences of drugs, and the hidden features of diseases store the attributes of diseases. What our model does is to retrieve the implicit characteristics of drugs and diseases based on the historical links of drugs-diseases and also the auxiliary information. By matching the drug hidden feature with the hidden feature of the disease, the probability that the drug can treat the disease can be obtained.

Several relevant definitions are given to facilitate the interpretation of the ANMF model.

### **Definition 1**

(**Drug-Disease relationship matrix**) *R* represents the drug-disease relationship matrix, where $R\in \mathbb {R}^{m\times n}$, *m* is the total number of drugs, and *n* is the total number of diseases. If drug *i* can treat disease *j*, then *R*[*i*][*j*] will be set to one, else will be set to zero.

### **Definition 2**

*DrugSim* represents the drug similarity matrix, where the value of *DrugSim*[*i*][*j*] indicates the degree of similarity between drug *i* and drug *j*, *DrugSim*_*i*∗_=[*DrugSim*_*i*1_,*DrugSim*_*i*2_…*DrugSim*_*im*_] represents the similarity vector between drug *i* and all drugs in the dataset. *DiseaseSim* represents the disease similarity matrix; where the value of DiseaseSim[i][j] denotes the degree of similarity between disease *i* and disease *j*, *DiseaseSim*_*j*∗_=[*DiseaseSim*_*j*1_,*DiseaseSim*_*j*2_…*DiseaseSim*_*jn*_] represents the vector of similarity between disease *j* and all diseases in the dataset.

### Datasets

There are two datasets used in the paper, the Gottlieb dataset [21] contains 593 drugs registered in DrugBank[22], 313 diseases listed in the Online Mendelian Inheritance in Man database (OMIM) [23] and 1933 validated drug-disease associations in total. The summary of the Gottlieb dataset is shown in Table 1.

**Table 1:** Statistics of the Gottlieb dataset

Dataset	Drugs	Diseases	Interactions	Sparsity
Gottlieb	593	313	1933	1.041×10^−2^

We performed additional experiments on the Cdataset [14]. The Cdataset contains 409 drugs registered in DrugBank [22], 663 diseases recorded in the OMIM database [23] and 2532 validated drug-disease associations. See Table 2 for details.

**Table 2:** Statistics of the Cdataset

Dataset	Drugs	Diseases	Interactions	Sparsity
Cdataset	409	663	2532	9.337×10^−3^

Here, drug similarities are calculated via the Chemical Development Kit (CDK) [24] based on Simplified Molecular Input Line Entry Specification (SMILES) [25]. Pairwise drug similarity and chemical structures are denoted as the Tanimoto score of their 2D chemical patterns. The similarities between diseases are obtained from MimMiner [26], which estimates the degree of pairwise disease similarity via text mining their medical descriptions information in the OMIM database. All of the above information can be obtained from [14].

### Hidden feature mining

In recent years, deep learning proved to be efficient in discovering high-level hidden representations from various raw input data. Various algorithms used the auxiliary information to deal with data sparsity in the field of recommendation systems. Therefore, inspired by the Additional Denoising Autoencoder (ADAE) [18] model from the recommendation systems field, we combined drug similarity, disease similarity, and deep learning to extract the hidden features of drugs and diseases.

The upper part of Fig. 1 shows the process of extracting the hidden feature of drug *i*. $s_{i}^{drug}=${ *R*_*i*1_,*R*_*i*2_,…*R*_*in*_} which is generated by the given drug-disease relation matrix *R*, where $s_{i}^{drug}$ that represents the relationship between drug *i* and all other diseases. Adding Gaussian noise to $s_{i}^{drug}$ and *DrugSim*_*i*∗_ respectively to produce $\tilde {s}_{i}^{drug}$ and $\tilde {DrugSim}_{i*}$. Inputting $\tilde {s}_{i}^{drug}$ and $\tilde {DrugSim}_{i*}$ as the original information and auxiliary information when performing the following described encoding and decoding operation.

First, the encoding procedure described by formula (1) is performed, where *drug*_*i*_ is the hidden feature of drug *i*, *g* represents an arbitrary activation function, *W*_1_ and *V*_1_ represent the weight parameters, and *b*_*drug*_ denotes the bias parameter.

1 $$ drug_{i}= g\left(W_{1}\tilde{s}_{i}^{drug}+V_{1}\tilde{DrugSim}_{i*}+b_{drug}\right)   $$

The decoding operation is performed by using formula (2). The objective is to generate the reconstructed value $\hat {s}_{i}^{drug}$ of $s_{i}^{drug}$, where *f* represents an arbitrary activation function, *W*_2_ represents the weight parameter and $b_{\hat {s}_{i}^{drug}}$ denotes the bias parameter.

2 $$ \hat{s}_{i}^{drug}=f\left(W_{2}drug_{i}+b_{\hat{s}_{i}^{drug}}\right)   $$

Likewise, formula (3) is also a decoding operation on *drug*_*i*_, and the purpose is to generate the reconstructed value $\hat {DrugSim}_{i*}$ of *DrugSim*_*i*∗_.

3 $$ \hat{DrugSim}_{i*}=f\left(V_{2}drug_{i}+b_{\hat{DrugSim}_{i*}}\right)   $$

As a result, the loss function caused by the above encoding and decoding operations is as shown in the formula (4). Where $\parallel s_{i}^{drug}-\hat {s}_{i}^{drug} \parallel ^{2}$ and $\parallel DrugSim_{i*}-\hat {DrugSim}_{i*} \parallel ^{2}$ represent the error caused by the input value and the reconstructed value, $\left (\sum _{l} \parallel W_l \parallel ^{2} +\parallel V_l \parallel ^{2}\right)$ controls the complexity of the model by allowing it to have a better generalization performance. *α* represents the equilibrium parameter and *λ* is the regularization parameter.

4 $$ {\begin{aligned} \arg\; \min_{\{W_{l}\},\{V_{l}\},\{b_{l}\}} \; \alpha \left\| s_{i}^{drug}-\hat{s}_{i}^{drug} \right\|^{2} & \,+\,(1-\alpha) \left\| DrugSim_{i*}-\hat{DrugSim}_{i*} \right\|^{2}\\ &\,+\,\lambda\left(\sum_{l} \parallel W_{l} \parallel^{2} +\parallel V_{l} \parallel^{2}\right)  \end{aligned}}  $$

By minimizing Eq.(4), the hidden feature of drug *i* can ultimately be obtained.

Similarly, the lower part of Fig. 1 shows the process of acquiring the hidden feature of disease *j*, which is theoretically the same procedure as extracting the hidden feature of drug *i*. The process substitutes the original information and auxiliary information with $s_{j}^{disease}$ and *Disease*_*j*∗_, where $s_{j}^{disease}=\{R_{1j},R_{2j},\dots R_{mj}\}$ represents the relationship between disease *j* and all other drugs.

### Generate predicted value

Through the above-described steps, we managed to acquire the hidden feature of drug *i* and the hidden feature of disease *j* respectively. The traditional matrix factorization model allows us to perform the inner product operation on *drug*_*i*_ and *disease*_*j*_ to obtain the predicted value $\hat {r}_{ij}$, which represents the probability that drug *i* can treat disease *j*. However, the traditional matrix factorization model has the limitation of insufficient learning ability caused by the use of a fixed and straightforward inner product to estimate complex drug-disease interactions. The inner product operation does not take into account the weight relationship between factors, and cannot learn the complex associations between drugs and diseases.

In reference to the GMF model, the ANMF model uses the product operation of GMF instead of the inner product operation of the traditional matrix factorization model. Consequently, the ANMF model can learn the nonlinear relationship between drugs and diseases by introducing neuronal nodes and the nonlinear activation function, which improves the accuracy of the ANMF model. To do this, first calculate the elementwise product of the drug hidden feature and the disease hidden feature, and then input it into the single layer perceptron to obtain the predicted value. By introducing the neural network, the model can learn nonlinear drug-disease relationship and exhibit better learning and prediction ability. The ANMF model predicts the drug-disease relationship as presented formula (5):

5 $$ \hat{r}_{ij}=F_{out}\left(h^{T}\left(drug_{i}\bigodot disease_{j}\right)\right)   $$

Where *drug*_*i*_ and *disease*_*j*_ respectively represent the hidden features of drug *i* and disease *j* calculated by the ANMF model, $\bigodot $ is the elementwise product, *h* represents the weight parameter, *F*_*out*_ represents an arbitrary activation function and $\hat {r}_{ij}$ denotes the predicted value.

### ANMF Learning process

Now, we will define the general loss function of the ANMF model, and introduce how the model can learn the corresponding parameters. In general, the loss function of the ANMF includes two parts: the loss caused by extracting drug hidden features and disease hidden features as well as the loss between the predicted values and the target values.

The loss function of drug *i* hidden feature extraction is defined as shown in formula (6) :

6 $$ {\begin{aligned} LossOfDrug_{i}&=\alpha \left\| s^{drug}_{i}-\hat{s}^{drug}_{i}\right\|^{2}\\ &\quad+(1-\alpha)\left\| DrugSim_{i*}-\hat{DrugSim}_{i*}\right\|^{2}\\ &\quad+\lambda \left(\sum_{l}\parallel W_{l}\parallel^{2}+\parallel V_{l}\parallel^{2}\right)  \end{aligned}}  $$

Where, *W*_*l*_, *V*_*l*_ denote the weight parameters, *λ* denotes the regularization parameter and *α* represents the equilibrium parameter. Similarly, the loss function of disease *j* hidden feature extraction is defined as shown in formula (7):

7 $$ {\begin{aligned} LossOfDisease_{j}&=\beta \left\| s^{disease}_{j}-\hat{s}^{disease}_{j}\right\|^{2}\\ &\quad+(1-\beta)\left\| DiseaseSim_{j*}-\hat{DiseaseSim}_{j*}\right\|^{2}\\ &\quad+\delta\left(\sum_{d}\parallel W_{d}\parallel^{2}+\parallel V_{d}\parallel^{2}\right)  \end{aligned}}  $$

Where *W*_*d*_, *V*_*d*_ denote the model parameters, *δ* denotes the regularization parameter and *β* represents the equilibrium parameter. The loss between the predicted value and the target value is defined as shown in formula (8):

8 $$ LossOfPrediction_{i,j}=r_{ij}\log\hat{r}_{ij}+(1-r_{ij})\log(1-\hat{r}_{ij})   $$

Where *r*_*ij*_ denotes the target value and $\hat {r}_{ij}$ denotes the predicted value.

As a result, the general loss function for the training model is presented in formula (9):

9 $$ {\begin{aligned} Loss&=\sum_{(i,j)\in R^{+}\cup R^{-}}LossOfPrediction_{i,j}+\varphi LossOfDrug_{i}\\ &\quad+\psi LossOfDisease_{j}  \end{aligned}}  $$

where *R*^+^ denotes a set of positive instances and *R*^−^ denotes a set of negative instances, which can all be (or sampled from) unobserved drug-disease interactions. Where *φ* and *ψ* denote for the hyperparameters of the loss function.

As shown formula (6), formula (7) and formula (8), the mathematical formulas for LossOfPrediction, LossOfDrug, and LossOfDisease share similar fragments, namely *drug*_*i*_ and *disisease*_*j*_. In other words, the parameters contained in *drug*_*i*_ and *disisease*_*j*_ are shared by two steps of mining hidden feature and generating predicted value. It is these shared parameters that serve as a bridge between the two steps. Moreover, parameters are trained simultaneously. Thus, the information contained is orthogonal. This also ensures that there is no overlap in information in formula (9). And enabling our model to simultaneously learn effective hidden features, and capture drug and disease similarity and relationship.

The parameters of the ANMF model can be learned by minimizing formula (9), using the stochastic gradient descent method(SGD).

## Results

In this section, we will systematically evaluate the performance of the ANMF model using the Gottlieb dataset [21]. First, the evaluation metrics used in this study will be introduced. Next, the performance of the ANMF model under various parameter settings will be compared to find the optimal parameter settings. And we will survey the ANMF model’s performance with several state-of-the-art algorithms by referring to the evaluation metrics previously described, including new drug scenario. To further validate the robustness of the ANMF model, further experiments on the Cdataset [14] will be presented.

### Evaluation metrics

For a systematical evaluation of the ANMF model’s performance in comparison to other approaches, we adopted ten-fold cross validation (10-CV). To implement ten-fold cross validation, we randomly split all verified drug-disease associations in the dataset into ten equal-sized subsets, and all non-verified associations are considered as candidate associations. In each fold, we considered one subset as the test set, while the combined remaining nine subsets served as the training set. All candidate associations were then added to the test set. After the ANMF model training is completed, the associations in the test set will get a corresponding score.

In this study, we denoted the verified drug-disease associations as positive samples, while the remaining unverified associations were considered as negative samples. For each specific threshold, we calculate the corresponding true positive (TP), true negative (TN), false positive (FP), and false negative (FN) values. If a test association’s corresponding score is greater than the threshold, it was labeled as a positive sample. Else, it was considered as a negative sample. Hence, TP and TN values characterized the number of positive and negative samples correctly identified. FP and FN values denoted the number of positive and negative samples misidentified. By regulating the threshold, we were able to obtain the True Positive Rate (TPR) and False Positive Rate (FPR). Finally, the AUC (Area Under Curve) value was acquired by drawing the Receiver Operating Characteristic (ROC) curve. Moreover, this study also used AUPR (Area Under Precision-Recall Curve) as the second evaluation indicator. Because AUC measure does not capture all aspects of the model’s performance, adding the AUPR measure can more fully reflect the true performance of the model. The Hit Ratio (HR) evaluation indicator was also used in this study. Intuitively, HR measures the presence of the positive samples within the top N. And HR@n means Hit Ratio with cut offs at n.

### Parameters setting

The main parameters that the ANMF model needs to set are the hidden feature dimension, and the number of negative sampling. This is due to the fact that, the size of the hidden feature vector controls the complexity of the ANMF model, while the number of negative sampling controls the generalization capabilities of the proposed model. Hence, two experiments are conducted for evaluating the performance of the model under both different dimension values of hidden feature vector and different negative sample sizes.

All hyperparameters are set as follows: In order to reduce the amount of calculation, *φ* and *ψ* in Eq. (9) were set to 0.5, by default. Similar to [16], we use a masking noise with a noise level of 0.3 to get the corrupted inputs from the raw inputs. The rest of hyperparameters are tuned according to the validation set. The validation set is formed by holding out one interaction per drug from the training set. We perform a grid search over *α* in formula (6) from {0.1,0.3,0.5,0.7,0.9} and *β* in formula (7) terms {0.1,0.3,0.5,0.7,0.9}. In addition, we varied regularization parameters *λ* and *δ* from {0.1,0.01,0.001}. Moreover, the dimension of the hidden feature varies from {16,32,64,128,256} and the number of negative sampling varies from {1,5,10,15,20}. Finally, we set *α*, *β*, *λ*, *δ*, the dimension of the hidden feature and the number of negative sampling to 0.7, 0.3, 0.001, 0.01, 128 and 10 according to the performance of the model on the validation set.

#### The dimension of hidden feature

Since it controls the complexity of the model, the dimension of the hidden feature vector is a very important parameter for the ANMF model. If the dimension of hidden feature vector was set to a large value, the model will likely to over-fit. But if the dimension was set to a small value, the model will not be able to learn the high-level association between drugs and diseases. Thus, the following experiment was preformed to observe the performance of the ANMF model in different settings, and to have a clear understanding in regards to the appropriate dimension value that required to be set for the hidden feature vector.

**Fig. 2:**
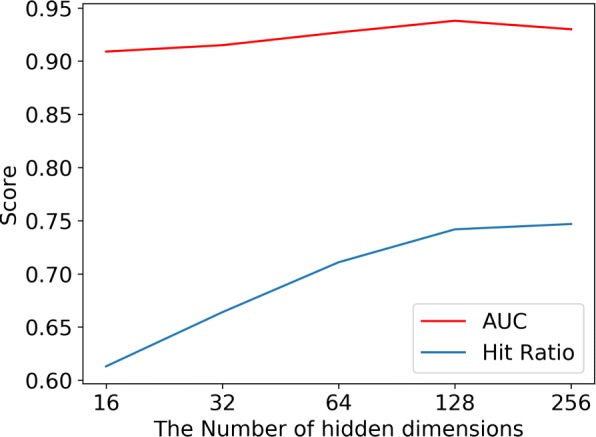
The performance of ANMF model under different hidden feature dimensions

Figure 2 illustrates the performance of the ANMF model on the Gottlieb dataset under different dimension values of the hidden feature vector. We can observe that there is a steady improvement as the dimension of the hidden feature vector increases, where a dimension value of 128 shows a peak in HR@10 performance, followed by a degradation potentially due to overfitting. As the dimension grows, the model’s AUC value and Hit Ratio value increases. This scenario shows that the ANMF model can capture more complex associations between drugs and diseases as the dimension increases. However, the AUC value has a downward trend as the dimension of value varies in the range [128,256], this confirms that the model tends to over-fit when the dimension of the hidden feature vector is too large. The larger the dimension value of the hidden features, the more complex the model will be. According to Occam’s razor law, among models with the same effect, a model with a lower complexity should be selected. So 128 was chosen as the appropriate dimension parameter value for the ANMF model.

#### Defining the number of negative sampling

The inclusion of the negative samples is a crucial step to the ANMF model. In this study, we refer to the idea of the negative sampling techniques in natural language processing [20] to enhance the training set. For each validated drug-disease association in the training set, we randomly take in N associations that have not been verified as negative samples into the training set. Since the number of positive samples, in reality, is much smaller than the number of negative samples, the above approach is desirable. However, Negative sampling is risky. The greater the number of negative sampling, the more it will increase the probability of forming a wrong negative sample or forcing the unknown positives to be considered negative. Therefore, we conducted this experiment to observe the performance of the model at different numbers of negative sampling.

**Fig. 3:**
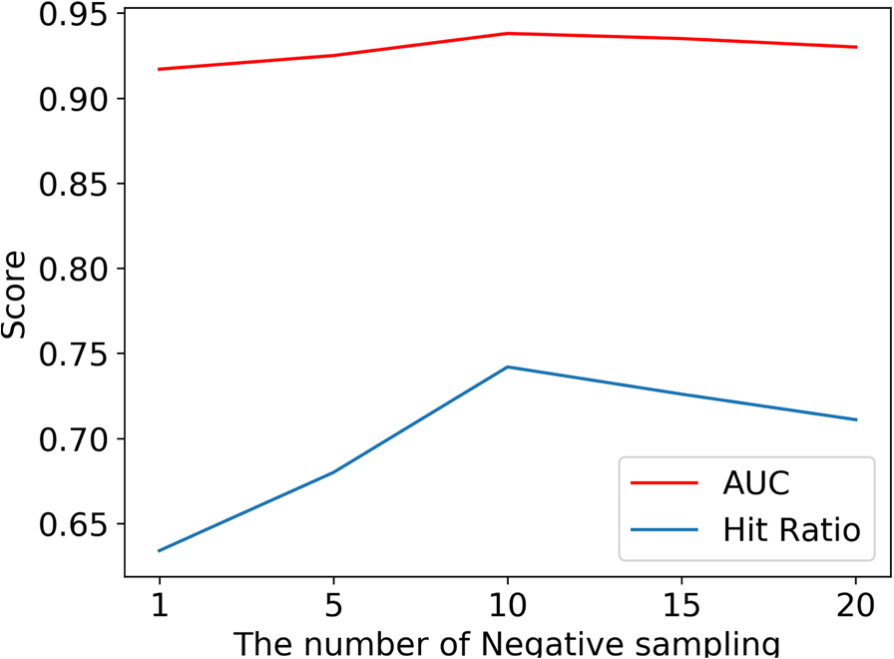
The performance of ANMF model under different negative sampling number

The abscissa calculated from of Fig. 3 represents the value of N. Figure 3 illustrates the performance of the ANMF model on the Gottlieb dataset when the negative samples value varies from [1,20]. We can observe a steady improvement as the number of negative samples grows. This scenario clearly demonstrates that using negative sampling techniques to enrich the training set is effective. However, when the value of N ranges from 10 to 20, both the AUC and the Hit Ratio values tend to decrease, which shows that wrong negative samples were forming as the value of N is increasing. According to the above experiment, we set the appropriate value of N to 10.

The experimental results clearly demonstrates that the negative sampling technique has a certain degree of improvement on the prediction effect and generalization performance of the model, which explains the effectiveness of the negative sampling technique to some extent.

### Baselines and comparison

With the aim of evaluating the performance of the proposed ANMF model, we will compare it with the current three most advanced models, DRRS [14], GMF [19] and HGBI [9].

DRRS is currently considered to be one of the best algorithms in the field of drug repositioning. This algorithm works by constructing a heterogeneous network via exploiting the drug-disease relationships, drug similarity and disease similarity. It then implements a fast Singular Value Thresholding (SVT) algorithm to complete the drug-disease adjacency matrix with predicted scores for previously unknown drug-disease associations.

GMF is a matrix decomposition model, in which neural networks and matrix decomposition are combined to enable the capturing of the nonlinear relationships between drugs and diseases. In other sense, the GMF model is an ANMF model without an auxiliary information version.

HGBI is introduced based on the guilt-by-association principle, as an intuitive interpretation of information flow on the heterogeneous graph. The parameters setting for the above mentioned methods are all established according to their corresponding literature. The overall performance of all methods is evaluated by applying the ten-fold cross validation technique (10-CV) specified in “Evaluation metrics” section.

The experiment results in terms of AUC, AUPR and Hit Ratio values are illustrated in Table 3. As clearly shown by the experimental results of Table 3, the proposed ANMF model outperforms other competitive methods in terms of AUC value. More specifically, the ANMF has an AUC value of 0.938, while DRRS, GMF, and HGBI yield results of 0.93, 0.88, and 0.829, respectively. Moreover, in terms of AUPR value, the ANMF model achieved the highest value of 0.347, while DRRS, GMF, and HGBI have results of 0.292, 0.281, and 0.16, respectively. Next, we compared the performance of the ANMF model with the other three models in terms of Hit Ratio value. The proposed ANMF model surpasses other models in regards to HR@1, HR@5, and HR@10. Furthermore, in the case of HR@10, our proposed ANMF model has a Hit Ratio value of 74.2%, while DRRS, GMF, and HGBI have 72.7%, 61.9%, and 59.3%, respectively.

**Table 3:** Prediction results of different methods on Gottlieb dataset

Method name	AUC	AUPR	HR@1	HR@5	HR@10
ANMF	0.938	0.347	47.9%	61.3%	74.2%
DRRS	0.93	0.292	45.9%	53.1%	72.7%
GMF	0.88	0.281	35.1%	48.5%	61.9%
HGBI	0.829	0.16	33%	45.4%	59.3%

### Predicting indications for new drugs

The ANMF model can also be used for drugs without previously known disease associations. One hundred seventy-one drugs in the Gottlieb data set only has one known drug-disease association. In this case, we will be taking 171 known association as the test set, the remaining verified associations are considered as the training set. The evaluation metrics are AUC value, AUPR value and Hit Ratio. The experimental results in terms of AUC value, AUPR value and Hit Ratio are presented in Table 4.

**Table 4:** Prediction results of different methods for new drug on Gottlieb dataset

Method name	AUC	AUPR	HR@1	HR@5	HR@10
ANMF	0.859	0.161	28.1%	34.5%	46.2%
DRRS	0.824	0.107	28.1%	30.4%	39.2%
GMF	0.813	0.106	18.1%	19.3%	21.1%
HGBI	0.746	0.065	9%	14%	24.6%

As shown in Table 4, the performance of our proposed ANMF model is superior to other competitive methods regarding AUC value. More specifically, the AUC value of the ANMF model is 0.859, while the results of DRRS, GMF, and HGBI are 0.824, 0.813, and 0.746, respectively. Moreover, in terms of AUPR value, the ANMF model achieved the highest value of 0.161, while the results of DRRS, GMF, and HGBI are 0.107, 0.106, and 0.065, respectively.

Now we turn to the comparison of the ANMF model performance with the other previously mentioned models in terms of Hit Ratio value. As likewise shown in the experimental results in Table 4, the proposed ANMF model outperforms other models. In regards to the HR@1 case, the DRRS model has the same hit ratio as the ANMF. However, in the case of HR@5 and HR@10, the hit ratio value of the ANMF model is superior to those of the other examined models. For instance, in the case of HR@10, the Hit Ratio value of the ANMF model is 46.2%, while the Hit Ratio values of DRRS, GMF, and HGBI is 39.2%, 21.1%, and 24.6% respectively.

### Validation on the Cdataset

To further validate the robustness of the proposed ANMF model, we performed additional experiments on the Cdataset [14]. The evaluation metrics used in this validation phase experiment are the same as the ones mentioned in “Evaluation metrics” section. The hidden features dimension and the number of negative sampling were set to 256, and 10, respectively. Other hyperparameter settings remain the same.

In terms of predicting known associations, the results of this experiment portrayed in Table 5 show that the ANMF model measured an AUC value of 0.952, a superior outcome when compared to the AUC values that of DRRS, GMF, and HGBI which were 0.947, 0.915, and 0.858 respectively. Moreover, in terms of AUPR value, the ANMF model achieved the highest value of 0.394. Concerning the Hit Ratio value, the ANMF model similarly performed better than the other models in the case of HR@1, HR@5 and HR@10. For instance, in the case of HR@10, the Hit Ratio value of the ANMF model is 76.3%, while the DRRS, GMF, and HGBI models measured Hit Ratio values of 70.1%, 56.3%, and 55.1% respectively.

**Table 5:** Prediction results of different methods on Cdataset

Method name	AUC	AUPR	HR@1	HR@5	HR@10
ANMF	0.952	0.394	42.1%	65.1%	76.3%
DRRS	0.947	0.351	32.3%	59%	70.1%
GMF	0.915	0.337	25.4%	39.7%	56.3%
HGBI	0.858	0.204	26.7%	37.1%	55.1%

According to the results in Table 6, the ANMF model likewise outperformed the previously mentioned models in predicting new drugs with an AUC value of 0.857, as opposed to 0.824 for DRRS, 0.798 for GMF, and 0.732 for HGBI. Moreover, in terms of AUPR value, the ANMF model achieved the highest value of 0.097. In terms of Hit Ratio value, the ANMF model measured a lower value than of the DRRS model for the HR@1 value, possibly because the Cdatasets is sparse. However, in the case of HR@5 and HR@10, the performance exceeded other models. For example, in the case of HR@10, the Hit Ratio value of ANMF is 37.3%, while that of DRRS, GMF, and HGBI were 35%, 26% and 26% respectively.

**Table 6:** Prediction results of different methods for new drug on Cdataset

Method name	AUC	AUPR	HR@1	HR@5	HR@10
ANMF	0.857	0.097	19.2%	33.3%	37.3%
DRRS	0.824	0.084	25.4%	30.5%	35%
GMF	0.798	0.071	13.6%	17%	26%
HGBI	0.732	0.022	11.3%	21.5%	26%

## Discussion

Through experiments performed on two real-world datasets, we managed to demonstrate that the proposed ANMF model outperformed other portrayed methods, and displayed significant performance enhancements. For the Gottlieb dataset, the AUC, AUPR and Hit Ratio measured values were 0.938, 0.347 and 74.2% respectively. And the model’s predictive performance on the Cdataset was 0.952 for the AUC value, 0.394 for AUPR value and 76.3% for the Hit Ratio value. The above-declared findings are all superior to their counterparts among other surveyed algorithms. Furthermore, we can deduce that using negative sampling techniques to enrich the training set showed to be effective through the performed experiments in “Defining the number of negative sampling” section.

Moreover, integrate assistance information to assist the model in overcoming the challenges of data sparsity. By comparing the performance of the ANMF model and the GMF model, which is an ANMF model with no auxiliary information version, the ANMF model outperforms the GMF model both in terms of AUC, AUPR and Hit Ratio values on two common data sets. And as the sparseness of the data set increases, the gap between the performance of the ANMF and the GMF model also increases. This result demonstrates the correctness of our initial assumption that integrating auxiliary information can overcome the sparseness of the data to a certain extent.

## Conclusion

As a vital and lucrative technology to discover new applications of old drugs, computational drug repositioning has been receiving growing attention from both the industry and academia. In this paper, we proposed an Additional Neural Matrix Factorization (ANMF) model for computational drug repositioning. The ANMF model combined deep learning representation with the nonlinear matrix factorization technique, to resolve the problems of data sparsity and insufficient learning ability. Furthermore, the negative sampling technique was employed to overcome the issue of model overfitting. Exhaustive experiments under multiple configurations demonstrated significant improvements over related competitive benchmarks. However, we believe that improvements can be made to the ANMF model in the future research. This study only makes use of drug similarity and disease similarity, and the attribute information of drugs and diseases is not limited to these two features. Furthermore, the ANMF model only uses a single-layer perceptron, which is the simplest deep learning model. For future work, using a complex deep learning model along with other auxiliary information to learn drug-disease relationship promises to deliver far improved results.

## Data Availability

The datasets and source code that support the findings of this study are available in https://github.com/MortySn/ANMF.

## Abbreviations

ADAE: Additional stacked denoising autoencoder
ANMF: Additional neural matrix factorization
AUC: Area under curve
AUPR: Area under precision-recall curve
CDK: Chemical development kit
DRRS: Drug repositioning recommendation system
FDA: The US food and drug administration
FN: False negative
FP: False positive
FPR: False positive rate
GMF: Generalized matrix factorization
HGBI: Heterogeneous graph based inference
HR: Hit ratio
HR@n: Hit ratio with cut offs at n
NMF: Non-negative matrix factorization
OMIM: Online mendelian inheritance in man
ROC: Receiver operating characteristic
SGD: Stochastic gradient descent method
SMILES: Simplified molecular input line entry specification
SVT: Fast singular value thresholding algorithm
TN: True negative
TP: True positive
TPR: True positive rate
10-CV: Ten-fold cross validation.
